# Medical gases in respiratory diseases: ozone, argon, and nitric oxide as game-changers in therapeutics

**DOI:** 10.3389/fmed.2025.1598798

**Published:** 2025-08-22

**Authors:** Lizhen Chen, Qi Cai, Pengfei Zheng

**Affiliations:** ^1^Department of Pharmacy, The First Hospital of Putian City, Putian, Fujian, China; ^2^College of Environmental and Biological Engineering, Putian University, Putian, Fujian, China; ^3^School of Medicine, Nankai University, Tianjin, China; ^4^School of Computer Science and Technology, University of Science and Technology of China, Hefei, Anhui, China

**Keywords:** respiratory tract diseases, ozone, argon, nitric oxide, COVID-19

## Abstract

Respiratory diseases pose a significant global health burden, prompting the exploration of novel therapeutic strategies. This narrative review consolidates existing knowledge and critically examines the evolving role of medical gases, ozone, argon, and nitric oxide (NO), in respiratory medicine. Based on recent literature, it highlights how these gases, originally used for their physicochemical properties, have now undergone a “functional crossover,” revealing their broad therapeutic potential. Analysis of available evidence indicates Ozone exhibits dual mechanisms: redox balance regulation and antimicrobial effects, demonstrating efficacy in COVID-19 pneumonia and hospital disinfection. Argon, when delivered through cold atmospheric plasma jets (CAPPJ), provides broad-spectrum antibacterial effects and targeted treatment for bronchopleural fistulas. NO, beyond its vasodilatory role, is now a dynamic tool for airway inflammation monitoring and precision asthma management. However, challenges persist, including optimizing therapeutic windows, standardizing treatment protocols, and assessing long-term safety and efficacy. Future directions emphasize precision medicine, incorporating biomarkers, AI-driven diagnostics, and combination therapies to overcome current challenges and unlock the full potential of medical gases in treating respiratory diseases.

## 1 Introduction

Respiratory diseases pose a significant global public health challenge, with their burden continuing to rise (1, 2). In 2019, pneumonia was responsible for nearly 2.5 million deaths worldwide, making it the fourth leading cause of death (3). The age-standardized mortality rate for chronic obstructive pulmonary disease (COPD) stood at 41.9 per 100,000, contributing to 5.7% of all deaths (4). Bronchial asthma affects 339 million people globally, with 5–10% of cases classified as severe and often resistant to treatment (5). Acute respiratory distress syndrome (ARDS), a common complication in critically ill patients, continues to carry a high in-hospital mortality rate of 35–46%, with worse outcomes particularly among the elderly and those with multiple infections (6). While traditional treatments like bronchodilators can relieve airway spasms, they lack specificity when it comes to addressing virus-bacterial co-infections. Glucocorticoids, though effective as anti-inflammatories, can worsen oxidative stress and damage mitochondrial function in airway epithelial cells. Mechanical ventilation, while essential for maintaining oxygenation, is associated with risks of ventilator-induced lung injury and barotrauma. The pathogen spectrum is notably age-dependent: *respiratory syncytial virus* (RSV) is most prevalent in children (21.3%), whereas *Pseudomonas aeruginosa* is more common in the elderly (15.37%) (7). However, existing treatment strategies have yet to incorporate precise, age-specific management approaches. Traditional therapies also fail to address crucial pathological processes, such as immune dysregulation and epithelial barrier repair. Around 22.8% of patients with bacterial pneumonia fail to respond to antibiotic treatment (8). These challenges underscore the pressing need for novel therapeutic approaches, including targeted modulation of inflammatory pathways, microbiome interventions, and the use of biologics.

The medical use of gases dates back to the introduction of oxygen therapy in the 18th century, but significant advances occurred in the late 20th century with the discovery of nitric oxide’s (NO) vasodilatory effects. In 1998, inhaled NO therapy was approved by the FDA for persistent pulmonary hypertension of the newborn, marking the formal entry of gas therapy into the era of evidence-based medicine. Historically, medical gases have been used based on their physicochemical properties: ozone is employed for disinfection due to its strong oxidative capacity; argon, being inert, is used in cryoablation procedures; and NO acts as a selective pulmonary vasodilator. However, these applications were traditionally limited to single-function roles until the last decade, when the convergence of molecular biology and materials science propelled medical gases into a new era of “functional crossover.”

Recent studies have underscored the multifaceted benefits of medical gases in treating respiratory diseases. Argon, through the active species generated by cold atmospheric pressure plasma jets (CAPPJ), exhibits broad-spectrum antibacterial properties. In addition, its core-shell nanocomposite materials enhance the sensitivity of NO sensing down to parts-per-billion (ppb) levels, providing a promising approach for monitoring airway inflammation (9, 10). Ozone therapy has expanded beyond its traditional disinfection role; by upregulating antioxidant enzyme systems like superoxide dismutase (SOD) and glutathione peroxidase (GPx), it significantly reduces systemic inflammation and immunothrombosis in COVID-19 patients (11). NO has evolved from a basic vasodilator to a dynamic tool for monitoring airway inflammation. The detection of fractional exhaled NO (FeNO), in combination with bronchoalveolar lavage fluid analysis, allows for precise identification of Th2 inflammatory phenotypes, guiding the personalized application of biologics (12). These advances have led to three major paradigms in the crossover application of medical gases: Argon plasma coagulation (APC) technology, which combines thermal and non-thermal effects for minimally invasive and precise treatment in bronchopleural fistula repair (13); ozone autohemotherapy (O3-MAH), which induces immune tolerance and mitochondrial biogenesis, providing organ-protective effects in ARDS patients (14); and NO nano-delivery systems, combined with artificial intelligence (AI) algorithms, which are advancing the development of phenotype-guided precision drug delivery for asthma (15).

In this context, medical gases such as ozone, argon, and NO are emerging as promising therapeutic agents with complex mechanisms of action and expanding clinical relevance. This narrative review aims to critically examine the evolving landscape of medical gas therapy in respiratory diseases. We synthesize current literature to trace the development of these gases from basic physicochemical tools to multifunctional therapeutic agents. The review explores their mechanisms of action, evaluates emerging data from preclinical and clinical studies, and identifies key challenges, including knowledge gaps and technological limitations, which must be addressed to support their broader clinical application. Through this analysis, we aim to highlight future research directions that may unlock the full therapeutic potential of medical gases in respiratory care.

## 2 Ozone therapy for respiratory diseases

### 2.1 Dual mechanisms of action of ozone

In respiratory therapeutics, ozone exerts a dual mechanism of action, modulating redox balance while demonstrating potent antimicrobial effects. Recent mechanistic studies indicate that endogenous ozone plays a pivotal role in maintaining cellular redox homeostasis. Fernández et al. (16) demonstrated that medical ozone effectively restores redox balance by activating key antioxidant enzymes, such as glutathione peroxidase, thereby interrupting the cascade of oxidative damage. This mechanism has shown particular promise in preventing and managing oxidative stress-related conditions, especially among elderly populations. Importantly, the biological effects of ozone are highly context-dependent. Wu et al. (17) reported that co-exposure to ozone and fine particulate matter (PM2.5) synergistically activates the TRPV1 channel and NF-κB pathway, promoting neutrophil infiltration and elevated pro-inflammatory cytokine release. These effects contribute to small airway dysfunction and worsening asthma symptoms, underscoring the need for careful assessment of environmental pollutant exposure in clinical ozone applications.

Beyond its redox-modulating effects, ozone exhibits multi-level biological activity in anti-infective therapy. Preclinical studies have shown that ozone inactivates pathogens by disrupting viral capsid structures and compromising bacterial membrane integrity (18). Simultaneously, it enhances host immune defense through immunomodulatory mechanisms. Clinical evidence further supports its role: nebulized ozone therapy has been associated with a significant reduction in pneumonia incidence among COVID-19 patients. In a clinical study by Dengiz et al. (19), ozone inhalation at concentrations of 30–40 μg/mL reduced pneumonia occurrence by 37% and accelerated symptom improvement by 2.3 days. Systemic ozone therapy, particularly O3-MAH, has shown the ability to regulate immune responses and mitigate cytokine storms. Yousefi et al. (20) reported that O3-MAH reduced levels of inflammatory cytokines such as IL-6 and TNF-*α* by 42–58% and increased the CD4+/CD8 + ratio by 0.3–0.5 units, highlighting its potential as an adjunct therapy in severe COVID-19 cases.

Mechanistic insights have also reinforced the clinical relevance of ozone. In the treatment of COVID-19, ozone has progressed from an adjunctive option to a recognized component of standardized protocols. A multicenter randomized controlled trial (RCT) (21) demonstrated that O3-MAH combined with standard therapy improved diffusion capacity for carbon monoxide (DLCO) by 15.2% and increased the six-minute walk distance by 83 meters in patients with post-acute sequelae, outperforming standard therapy alone. Nevertheless, careful quality control is essential for clinical ozone application. Mahlooji et al. (22), through hemoglobin oligomerization assays, emphasized the importance of individualized ozone concentration control (within the 20–60 μg/mL range) to ensure therapeutic efficacy while minimizing oxidative damage to blood components. In hospital infection control, ozone-based disinfection has been adopted for both environmental surfaces and personal protective equipment. For example, an *in vitro* study validated an N95 mask disinfection protocol (20 ppm for 15 min) (23), achieving complete viral inactivation even after six repeated uses, an effective solution during medical supply shortages.

Despite promising advances, several scientific questions remain. At the molecular level, while regulatory elements such as NLRP12 (24) and FOXO3 (25) have been implicated in ozone responses, the dynamic interactions within their signaling networks under ozone exposure remain to be fully elucidated. Clinically, most existing studies focus on acute infections like COVID-19, with limited long-term data on chronic respiratory diseases such as COPD and asthma. Moreover, heterogeneity in ozone dosing, exposure duration, and delivery methods across studies has hindered the establishment of standardized treatment protocols. Addressing these limitations will require future research to define optimized treatment parameters and explore dose–response relationships using multi-omics approaches. Ultimately, such efforts will support the development of personalized ozone therapies aligned with precision medicine principles.

### 2.2 From therapy to toxicity—navigating respiratory health

Clinical evidence increasingly supports the adjunctive role of ozone therapy in managing COVID-19, particularly through enhancing oxygenation and modulating inflammatory responses. In a RCT, Serra et al. (26) reported that patients receiving ozone therapy experienced an average increase of 8.2% in oxygen saturation (SpO₂) compared to controls, along with significant reductions in C-reactive protein (CRP) and interleukin-6 (IL-6) levels by 62 and 57%, respectively. These effects may be attributed to ozone’s ability to activate erythrocyte membrane ATPase and enhance oxygen release from hemoglobin (21). Regarding clinical outcomes, a meta-analysis (27) found that ozone adjuvant therapy reduced mortality in hospitalized COVID-19 patients by 43% (OR = 0.57, 95% CI: 0.42–0.78) and lowered the need for mechanical ventilation by 31%. Preliminary case reports have also suggested potential benefits for patients with post-acute sequelae of COVID-19 (PASC); in one such report, combined major O₃-MAH therapy was associated with a 72.4% improvement in DLCO, though these findings require validation through controlled trials to establish clinical significance (13). However, further evidence highlights limitations. Another meta-analysis (28) indicated that although ozone therapy significantly reduced IL-6 (*p* = 0.032) and D-dimer levels (*p* = 0.047) in critically ill patients, it had no measurable effect on intensive care unit (ICU) admission rates or 28-day mortality. These mixed results underscore the need for larger phase III clinical trials to determine ozone’s efficacy and define its role within comprehensive treatment protocols.

In contrast to its therapeutic applications, ambient ozone exposure has well-documented adverse effects on respiratory health. Epidemiological studies have shown that long-term exposure to ozone concentrations exceeding 70 μg/m^3^ increases the risk of acute asthma exacerbations by 1.34 times (95% CI: 1.12–1.61) and accelerates the annual decline in Forced Expiratory Volume in 1 s (FEV₁) among COPD patients by approximately 18% (29). This impact varies by age group. A case–control study (30) found that children with asthma were 2.1 times more sensitive to ozone exposure than adults (*p* = 0.008), likely due to the incomplete development of airway epithelial barrier function during childhood. Mechanistic research has further revealed that ozone exposure elevates the airway oxidative stress index (OSI) to 4.7 ± 0.9, significantly above the safety threshold, by activating TRPV1 channels and suppressing the Nrf2 pathway (31). Notably, short-term peak ozone exposure (8-h average >100 μg/m^3^) has been linked to a 27% increase in pneumonia-related hospitalizations among COPD patients (95% CI: 15–39%), with this effect further amplified by co-exposure to PM2.5 (32).

From a public health policy perspective, ozone pollution control remains a complex challenge. Modeling studies (33) suggest that a 10% reduction in NOₓ emissions can decrease surface ozone concentrations by 3.2–4.7 μg/m^3^. However, shifts in the VOCs/NOₓ ratio may paradoxically lead to localized increases in ozone levels, underscoring the need for coordinated multi-pollutant control strategies. For example, the “nitrogen lifecycle management” approach (34), which combines high-efficiency industrial denitrification (>85%) with optimized agricultural nitrogen fertilizer use (20–30% reduction), has the potential to reduce ozone precursor emissions by 18–22%. In clinical settings, ozone-based disinfection technologies have been implemented for infection control. Automated ozone systems (35) can process up to 20,000 medical textile items in 8 h, achieving microbial inactivation rates exceeding 99.9%. However, a comparative study (36) has shown that traditional chemical disinfectants demonstrate superior inactivation efficacy against SARS-CoV-2 (log4.2–5.1) compared to ozone water treatment (log2.3), highlighting the need to tailor disinfection strategies to specific use cases.

Ongoing debate surrounds the therapeutic window and risk–benefit balance of ozone use. A cohort study (37) found that HIV-positive individuals exposed to ozone levels above 80 μg/m^3^ had a 1.89-fold increase in all-cause mortality (95% CI: 1.32–2.71), suggesting that ozone therapy may require stricter dosing control in immunocompromised populations. Additionally, Mahlooji et al. (22) reported inter-individual variability in ozone-induced hemoglobin oligomerization thresholds, ranging from 15 to 45 μg/mL, underscoring the importance of personalized dosing strategies. These findings are contributing to the development of new clinical guidelines, including the integration of biomarker-based monitoring systems, such as SOD2 activity and mitochondrial DNA copy number (38), to support precision treatment while minimizing risk.

### 2.3 From promising therapy to clinical reality—addressing the hurdles

Despite the unique therapeutic potential that ozone has demonstrated in the treatment of respiratory diseases, its clinical application continues to face several significant challenges. Chief among these is the control of toxicity risk. Exposure to high concentrations of ozone can lead to excessive oxidative stress, resulting in the disruption of cell membrane integrity and mitochondrial dysfunction through lipid peroxidation pathways (31). Animal studies have shown that ozone concentrations above 60 μg/mL increase malondialdehyde (MDA) levels in lung tissue by 3.2-fold while reducing SOD activity by 57%, compared to controls (39). This dose-dependent toxicity is particularly pronounced in patients with COPD. Moreover, ozone toxicity exhibits tissue-specific variability. For instance, bronchial epithelial cells are 2.3 times more sensitive to ozone than alveolar cells (38), providing a strong rationale for optimizing local delivery methods to reduce unintended tissue damage.

In terms of clinical efficacy, current studies are limited by substantial heterogeneity and design limitations. A meta-analysis (26) reported a 38% average improvement in the PaO₂/FiO₂ ratio following ozone treatment (95% CI: 29–47%); however, the median sample size across the included studies was only 62 cases, with most follow-up periods lasting less than 6 months. This limited sample size and short observation duration restrict the ability to evaluate long-term outcomes. In one longitudinal cohort study (21), patients who received ozone therapy experienced a 1.7% decrease in FEV₁/FVC after 12 months, compared to a 0.9% decline in the control group (*p* = 0.043), suggesting potential concerns regarding long-term pulmonary function. These findings emphasize the need for large-scale, multicenter RCTs to more accurately define ozone’s therapeutic window and safety profile. It is essential to distinguish between the biological effects of controlled medical ozone therapy and harmful environmental ozone exposure. A dose–response model developed by Zhang et al. (29) estimated that the therapeutic range of medical ozone (20–50 μg/mL) is separated from the toxic environmental range (80–120 μg/m^3^) by a safety margin of approximately 3 to 5 times. This finding provides theoretical support for carefully regulated clinical applications and highlights the importance of precision in dosing.

Balancing the medical benefits of ozone with the environmental risks of ozone pollution presents a core challenge for future development. On the environmental side, reducing nitrogen oxide (NOₓ) emissions has been shown to decrease ozone-related asthma hospitalizations. However, the increasing clinical demand for ozone therapy raises questions about its sustainable integration into healthcare. This paradox underscores the need for a comprehensive and interdisciplinary evaluation framework. The “ozone exposure equivalent” model (40), which incorporates environmental ozone levels, therapeutic dosing, and individual susceptibility parameters, offers a potential approach to assess the dynamic risk–benefit ratio. On the technical side, the development of nanoscale ozone probes (41) capable of detecting redox state changes in real time, with a sensitivity equivalent to 0.1 μmol/L of hydrogen peroxide, may allow for personalized and responsive dose adjustments. Combination therapies also hold promise: one animal study (42) demonstrated that co-administering Liver X Receptors (LXR) agonists with ozone reduced lipid peroxidation products by 42% while maintaining beneficial oxidative stress levels, offering a novel strategy to overcome toxicity limitations.

Recent progress in the understanding of ozone’s mechanisms and clinical relevance can be grouped into three areas: its biological action, its therapeutic role in respiratory diseases, and the assessment of its efficacy. Mechanistically, ozone has been shown to impact respiratory health by modulating the gut-lung axis microbiome, upregulating MMP12 expression, and influencing oxidative stress pathways. It also demonstrates antiviral properties against SARS-CoV-2. However, long-term exposure to environmental ozone is associated with an increased risk of childhood asthma and other respiratory conditions. Therapeutically, ozone has been found to upregulate host susceptibility genes such as ACE2, potentially increasing infection risk, while optimized dosing has been shown to inactivate viral RNA in the environment. Environmental factors like heat have been shown to exacerbate ozone’s effects on allergic rhinitis, and prenatal exposure has been linked to a higher risk of asthma in offspring. Although ozone therapy appears to reduce inflammatory markers and improve symptoms in COVID-19 patients, most available clinical evidence comes from case series, and its efficacy remains to be confirmed through larger trials.

Nevertheless, several pressing issues remain unresolved. At the molecular level, although ozone’s role in inducing oxidative stress and inflammatory responses is well established, the specific signaling pathways and regulatory targets have not been fully elucidated. In particular, the mechanisms underlying gender differences in ozone sensitivity are poorly understood, with limited data available to explain why women may exhibit greater clinical susceptibility. Moreover, the role of epigenetic factors, polymorphisms in metabolic enzymes, and other markers of individual vulnerability remains largely hypothetical. These knowledge gaps limit the development of personalized preventive strategies and hinder the formulation of individualized safety thresholds.

There is also a significant lack of high-quality clinical evidence to support the widespread adoption of ozone therapy in respiratory medicine. Most published studies are small in scale (fewer than 100 participants), have short follow-up periods (typically under 6 months), and suffer from poorly designed control groups. Notably, over 80% of studies have used non-randomized methodologies, resulting in considerable bias in the evaluation of treatment efficacy. Furthermore, most available safety data are based on short-term use, with insufficient information about long-term consequences such as progressive lung function decline or tumorigenic risk. This lack of robust evidence makes it difficult to include ozone therapy in clinical guidelines and impedes efforts to standardize treatment protocols.

In addition, understanding ozone’s interactions with other environmental factors remains an unresolved challenge. Existing models struggle to accurately capture the physicochemical interactions between ozone and co-pollutants such as PM2.5, especially under varying meteorological conditions. Although epidemiological research indicates that co-exposure to ozone and NOₓ leads to a nonlinear increase in health risks, laboratory data have yet to establish reliable dose–response models. Compounding this issue, climate change has introduced further volatility in ozone concentrations, thereby reducing the predictive power of conventional risk assessment tools. These complex dynamics undermine public health early warning systems and make it difficult to assess the effectiveness of environmental interventions.

To overcome these barriers, future research should prioritize three key areas. First, toxicity monitoring systems should be developed using oxidative stress biomarkers, enabling precision-guided drug delivery through real-time tracking of pathways such as Nrf2 and NF-κB (24). Second, integrated assessment tools for both medical and environmental ozone exposure must be created, incorporating parameters like background ozone concentrations, individual exposure history, and genetic susceptibility (30). Third, there is an urgent need to improve the standardization of medical ozone therapy. This includes establishing unified concentration calibration protocols (with error margins below ±5%), standardized operating procedures, and a global, multi-center clinical registry (22). These efforts will facilitate the evolution of ozone therapy from an empirical practice to a scientifically validated component of precision medicine, offering both controlled environmental risk and clinically meaningful outcomes.

## 3 Application of argon in the treatment of respiratory diseases

While ozone therapy offers dual therapeutic mechanisms, balancing redox status and exerting antimicrobial effects, its clinical translation is hindered by concerns over toxicity, a lack of standardized protocols, and insufficient high-quality, long-term evidence, particularly in the context of chronic respiratory diseases. In contrast, argon, initially regarded as an inert gas with limited physiological effects, is now gaining attention for its expanding role in respiratory medicine. Recent advancements in cold atmospheric plasma and APC have introduced new therapeutic pathways for addressing distinct clinical challenges in respiratory care.

Kabiolskiy et al. (9) demonstrated that inert gases, including argon, can modulate physiological systems by interacting with multiple cellular targets across the central nervous system, apoptotic cascades, and immune pathways. This pleiotropic nature gives argon a unique therapeutic potential in managing complex pathological states. CAPPJ technology, which relies on the combined action of high-energy electrons, ionized atoms, and ultraviolet photons (43), has shown promise in controlling microbial load in the respiratory tract. Although initial results are encouraging, further clinical validation is necessary. In the field of biosensing, a hemin-based nanocomposite with a core-shell structure (HNS-rGO), developed by Wang et al. (10) achieved high-sensitivity detection of NO by optimizing Fe–N₄ site exposure and improving physical stability. This platform provides a potential method for real-time monitoring of inflammatory activity in the respiratory system.

Clinically, APC has emerged as a core technique in bronchoscopic interventional therapy. For instance, Cao et al. (13) reported that combining endobronchial valve placement with localized argon stimulation significantly improved respiratory function in patients with complex bronchopleural fistulas. In rare pediatric cases, such as endobronchial inflammatory myofibroblastic tumors, early application of an APC-based regimen combined with celecoxib achieved long-term disease control (44). In critical care, argon therapy in conjunction with extracorporeal membrane oxygenation (ECMO) enabled the successful removal of obstructive endobronchial casts and reversed complete ventilatory failure (45). A multidisciplinary approach has also proven valuable. In one case involving a bronchial glomus tumor, combining APC with radical surgical resection resulted in 2 years of disease-free survival (46), highlighting the potential of integrated treatment models in managing complex airway tumors.

Recent studies have made notable strides across three key areas: the mechanisms behind argon therapy, its application in treating respiratory diseases, and the broader use of noble gases in medicine. In terms of argon therapy, research has uncovered new insights into its role in gas measurement, radiation-resistant materials, cellular organization, and plasma therapy. For example, studies have confirmed the clinical applicability of argon in measurement, identified mechanisms by which argon contributes to the radiation-resistant degradation of polypropylene, explored how the orderly arrangement of alveolar spheroids influences tissue morphogenesis, and revealed the role of argon plasma in treating viral infections and tumors through reactive oxygen species modulation. In respiratory disease treatment, the expanding use of bronchoscopic technologies has become a major research focus. This includes the integration of both hard and soft bronchoscopes for diagnostic and therapeutic purposes, personalized surgical approaches to address airway stenosis, the application of APC for tumor resection and hemostasis, and the combined use of cryoablation with immunotherapy. These advancements have significantly improved diagnostic capabilities, treatment outcomes, and patient prognosis. Regarding the medical application of inert gases, research is concentrating on optimizing processes and enhancing performance. Areas of interest include argon-shielded welding to minimize emissions, nitrogen plasma disinfection technologies to boost environmental sustainability, the regulatory effects of inert gases on the central nervous system, and validating the safety of cryoablation for tumor treatment. These innovations offer fresh perspectives for medical environmental protection and industrial applications.

Despite these advances, key challenges remain. Mechanistic studies are largely confined to *in vitro* models or animal research, with limited data on how argon modulates molecular pathways in the human body (9, 43). In diagnostics, traditional imaging lacks sensitivity for detecting early-stage lesions, and biomarker dynamics are not yet well integrated into diagnostic frameworks (47, 48). Clinically, many therapeutic protocols involving argon are supported by single-center or small-sample studies. Their long-term safety, effectiveness, and applicability to diverse populations remain uncertain and call for validation through large-scale, multicenter clinical trials (49). To address these challenges, future research should prioritize building integrated multi-omics platforms, developing AI-based intelligent diagnostic systems, and improving the evidence-based medicine system of treatment protocols through real-world studies.

## 4 Application of NO in the treatment of respiratory diseases

Argon-based therapies, including APC and cold plasma-derived applications, have secured a meaningful role in respiratory interventions and diagnostics. Unlike ozone, which relies on its strong oxidative reactivity, or argon, which acts primarily through plasma-mediated physical interactions, NO functions as a critical endogenous signaling molecule. Its evolution, from a selective pulmonary vasodilator to a non-invasive biomarker for airway inflammation (FeNO), and potentially a high-dose antimicrobial agent, illustrates the dynamic and versatile therapeutic potential of medical gases in managing respiratory diseases.

### 4.1 Biological functions of NO

At the molecular level, NO plays a central role in signaling regulation. For example, L-citrulline promotes NO synthesis, enhancing endothelial function and opening new avenues for therapeutic intervention in conditions like sickle cell disease and postoperative pulmonary hypertension (15). Notably, FeNO serves as a non-invasive biomarker of Th2-type inflammation, with concentration changes closely correlating with the degree of airway inflammation, making it particularly useful in the diagnosis and monitoring of allergic respiratory diseases such as asthma (12). In addition to its role in inflammation, NO exhibits antimicrobial effects at high concentrations. Inhaled NO (iNO) administered at doses exceeding 160 ppm has demonstrated the capacity to directly kill respiratory pathogens, offering a promising strategy for treating drug-resistant infections, a finding supported by meta-analytic evidence (50). Furthermore, the bidirectional vascular regulatory properties of NO are integral to its therapeutic use in persistent pulmonary hypertension of the newborn, where its selective pulmonary vasodilatory action substantially improves oxygenation status (51).

### 4.2 Addressing the spectrum of respiratory challenges

In the management of chronic airway diseases, dynamic FeNO monitoring represents a major step forward in precision medicine. Measuring FeNO200 at varied flow rates has proven effective for assessing peripheral airway and alveolar inflammation in COPD patients, enabling more precise corticosteroid titration (52). In severe asthma, case series have shown that single-inhaler triple therapy (ICS/LABA/LAMA) improves small airway function and reduces acute exacerbation frequency through synergistic effects (53). In critical care, iNO has been employed as a rescue therapy for refractory hypoxemia in ARDS. Although controlled studies suggest short-term benefit, broader clinical utility remains under investigation. Preliminary, uncontrolled reports have also indicated that iNO may optimize pulmonary hemodynamics in conditions like persistent pulmonary hypertension of the newborn (51, 54). Moreover, case-based evidence suggests that NO may improve ventilation-perfusion matching in complex scenarios such as dermatomyositis-associated interstitial pneumonia requiring mechanical ventilation (55). These findings are promising but require validation in larger, controlled trials.

### 4.3 Addressing gaps in evaluation and translation

Despite progress, major gaps persist in evaluating NO’s clinical efficacy. Most trials rely on short-term endpoints, lack standardized follow-up frameworks, and are limited by the absence of objective, biologically relevant indicators, such as comprehensive inflammatory profiles, which restrict comparability across studies. Additionally, precision medicine approaches have yet to fully integrate patient-specific genotypic and molecular endotypes. For example, polymorphisms like NOS2 rs10459953 remain underexplored in the context of individualized NO-based therapies (56). These gaps hinder the development of robust, targeted treatment strategies. From a health system perspective, translational challenges are considerable. Many primary care institutions lack the infrastructure and technical training required for safe iNO administration, leading to substantial disparities in access. Overcoming this will require the establishment of multi-center clinical databases, the development of AI-based predictive models for treatment response, and the design of portable, user-friendly NO detection devices to facilitate broader implementation and protocol standardization.

At the pharmacological level, NO has demonstrated wide-ranging effects. It plays a role in inhibiting SARS-CoV-2 replication, serves as a biomarker in pulmonary nodular disease, improves endothelial dysfunction, and enhances therapeutic delivery via nanocarriers. In respiratory medicine, the diagnostic and therapeutic utility of NO has been confirmed in diverse contexts, including COVID-19 oxygenation improvement, asthma phenotyping, and occupational asthma diagnosis. Advances in FeNO assay technologies and their integration with clinical parameters have significantly improved disease management. Moreover, emerging evidence points to the involvement of the TGF-*β*/iNOS pathway in tumor metastasis and suggests that NO-guided asthma protocols may offer cost-effective benefits. However, the clinical efficacy of iNO in ARDS remains controversial, and deeper understanding of tissue-specific NO metabolism is still needed. Although research has advanced from understanding basic mechanisms to exploring clinical applications, significant breakthroughs in personalized therapeutic strategies and precise monitoring techniques are still required, and several critical challenges remain to be addressed.

Despite a growing body of research linking NO to numerous disease processes, its precise molecular mechanisms remain incompletely understood. Most current studies are confined to specific signaling pathways or disease models, and there is a lack of systematic investigation into how NO dynamics vary across different tissue microenvironments. Particularly, the bidirectional regulation of NO and its concentration-dependent effects at different stages of disease progression remain poorly understood. Moreover, research on the interaction between NO and other bioactive molecules is limited, hindering a comprehensive understanding of its precise role in the development of complex diseases. This gap in foundational research directly impedes the creation of effective targeted interventions.

At present, there is no standardization of therapeutic dosages or treatment protocols, with significant variations in dosing methods and treatment durations across different studies, complicating comparisons of clinical outcomes. In terms of efficacy, most assessment tools rely on subjective symptom scores or individual biomarker tests, lacking an objective and comprehensive evaluation framework. Furthermore, individualized treatment regimens for patients with varying disease types and severities have yet to be established, making precision medicine difficult to implement. Most critically, insufficient data on the safety and tolerability of long-term treatment restricts its use in chronic disease management. These unresolved issues in clinical application urgently need to be addressed.

Current NO detection methods are not equipped for real-time, dynamic monitoring of physiological concentrations, particularly at specific tissue sites, where achieving accurate measurements remains technically challenging. The development of NO delivery systems also faces significant hurdles in achieving controlled release and targeted distribution. Existing platforms struggle with limited precision in regulating local drug concentrations and treatment durations. Safety concerns further complicate the use of NO therapy due to its complex dose–response relationship, where excessive concentrations can lead to toxicity, yet current technologies lack reliable methods for maintaining the precise therapeutic window.

## 5 Discussion and future perspectives

The exploration of medical gases, ozone, argon, and NO, in respiratory medicine marks a shift from single-function agents, typically selected for their basic physicochemical properties, to versatile therapeutic tools that engage complex biological pathways. Ozone harnesses its strong oxidizing capacity for redox modulation and antimicrobial effects. Argon’s therapeutic potential is increasingly recognized through the physical and chemical actions of plasma technologies like CAPPJ and APC. As an endogenous signaling molecule, NO offers a wide range of therapeutic possibilities, from vasodilation and inflammation monitoring to targeted antimicrobial action. The diverse mechanisms of action behind these gases highlight their “functional crossover,” providing strong support for their expanding roles in respiratory medicine. However, despite significant advancements, realizing the full potential of these therapies faces common challenges. These include the need for a deeper, multi-omics understanding of their mechanisms, extending beyond single pathways [e.g., interactions between ozone/LXRs (42) and NO/STAT6 (57) signaling], difficulties in clinical translation such as the standardization of protocols and ensuring long-term safety and efficacy, and the necessity for robust delivery and monitoring systems. Overcoming these challenges will require focused efforts. Looking ahead, three interconnected future directions are crucial for advancing medical gas therapies in respiratory diseases:

### 5.1 Advancing precision medicine through biomarker integration and AI

Given the heterogeneity of respiratory diseases and the variability in patient responses, a shift away from a one-size-fits-all approach is essential. A key future direction involves developing a comprehensive precision medicine framework that integrates validated biomarkers (58, 59). For example, FeNO already helps guide corticosteroid adjustments in asthma and COPD by reflecting Th2 inflammation. Similarly, for ozone therapies, dynamic monitoring of redox states (e.g., using nanoscale probes or assessing antioxidant enzyme activity like SOD) could enable personalized dosing within its narrow therapeutic window. For argon-based therapies like APC, identifying reliable markers of tissue response and therapeutic effect will be essential. Integrating genomic data, such as NOS2 gene polymorphisms that affect NO metabolism (60), can further refine patient stratification and dosing. Furthermore, AI offers powerful tools for analyzing complex, multidimensional datasets, including biomarkers, genomics, and clinical parameters. AI-driven predictive models could forecast individual treatment responses, optimize dosing regimens, and even design more efficient, adaptive clinical trials, thereby accelerating the development of personalized gas therapies.

### 5.2 Harnessing synergies through multimodal therapeutic strategies

Given the multifactorial nature of respiratory diseases, combining therapeutic modalities may yield better outcomes than monotherapies. Medical gases, with their diverse and complementary mechanisms, are well-suited to synergistic approaches. Preliminary data suggest benefits from combinations such as APC with iNO in complex airway disorders (61), ozone with antioxidants to balance oxidative effects, argon with endobronchial valve therapy for fistulas, and ozone autohemotherapy (O₃-MAH) with conventional COVID-19 regimens. Future research should systematically evaluate rational therapeutic combinations, not only among different medical gases but also alongside pharmaceutical agents, such as LXR agonists enhancing ozone’s anti-inflammatory activity. Additional synergies may arise from integrating gas therapy with pulmonary rehabilitation, personalized ventilatory strategies (62–64), neuromodulation techniques influencing respiratory control (65), and digital health interventions for remote monitoring and adherence support (66). Adopting a multimodal, systems-based perspective will be essential for addressing the complexity of respiratory conditions and optimizing patient-centered outcomes across diverse clinical settings.

### 5.3 Overcoming translational barriers with innovative delivery and monitoring

One of the main challenges preventing the widespread use of medical gases, particularly ozone and high-dose NO, is the difficulty in accurately controlling their concentration at the target site while minimizing systemic exposure and the risk of toxicity. Significant progress is needed in delivery methods and real-time monitoring technologies. Targeted delivery systems, such as nanocarriers for controlled NO release, have the potential to revolutionize treatment by enhancing local efficacy and reducing side effects. For argon, improving technologies like CAPPJ and APC to achieve greater precision in energy delivery is also crucial. At the same time, developing advanced monitoring platforms is essential, including highly sensitive, real-time sensors for both gas concentrations (potentially utilizing innovative nanocomposite materials) and physiological responses, possibly linked to Internet of Things (IoT) systems for continuous data capture. Intracellular monitoring tools, such as nanoscale ozone probes, could provide valuable insights into cellular effects and help adjust doses more accurately. The successful development of these technologies will be pivotal for achieving precise spatiotemporal control, optimizing the therapeutic window, ensuring patient safety, and ultimately translating promising gas therapies from bench to bedside.

While ozone, argon, and NO each present their own set of opportunities and challenges, their collective progress marks a major shift in respiratory treatments. By advancing precision medicine through the use of biomarkers and AI, exploring synergistic multimodal strategies, and developing innovative delivery and monitoring systems, the field can overcome current limitations. Ongoing collaboration between researchers, clinicians, engineers, and data scientists will be vital in unlocking the full potential of medical gases and ushering in a new era of “Precision Medicine 2.0” for patients with respiratory diseases.

## 6 Conclusion

**Figure 1:**
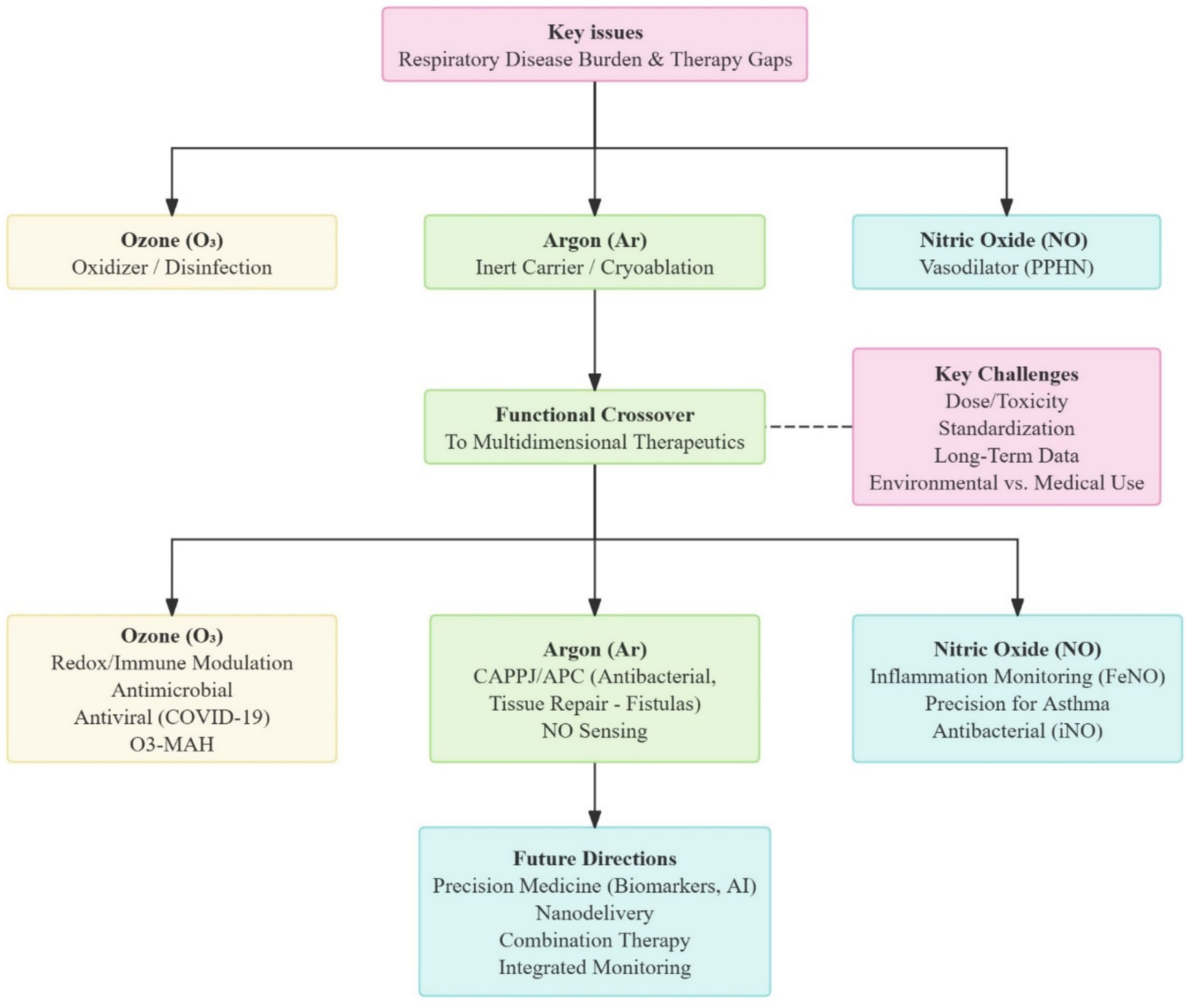
Conceptual diagram of medical gases in respiratory diseases. AI, Artificial Intelligence; APC, Argon Plasma Coagulation; CAPPJ, Cold Atmospheric Plasma Jet; COVID-19, Corona Virus Disease 2019; FeNO, Fractional Exhaled Nitric Oxide; iNO, Inhaled Nitric Oxide; O3-MAH, Ozone Major Autohemotherapy; PPHN, Persistent Pulmonary Hypertension of the Newborn.

From the redox regulation of ozone to the multimodal physical effects of argon, and the signaling network regulation of NO, medical gases have evolved from adjunctive tools with single functions to essential components within the diagnostic and therapeutic frameworks for respiratory diseases (Table 1; Figure 1). This transformative shift not only redefines the therapeutic approach of traditional respiratory medicine but also signals a profound change in future medical practice, from “molecular targeting” to “system regulation.”

**Table 1:** Comparison of medical gases in respiratory diseases.

Comparison factor	Ozone (O3)	Argon (Ar)	Nitric oxide (NO)
Primary mechanism	Redox modulation (↑antioxidant enzymes), Direct antimicrobial (disrupts viral capsid/bacterial membrane)	Physical/chemical effects via plasma (CAPPJ), Thermal & non-thermal effects (APC)	Endogenous signaling, Vasodilation, Biomarker of Th2 inflammation (FeNO), High-dose antimicrobial
Key technology	O3-MAH (Autohemotherapy), Nebulized inhalation, Ozone water/gas disinfection, Nanoscale probes (future)	APC, CAPPJ (Cold Atmospheric Plasma Jet), Carrier in cryoablation, Potential ECMO adjunct	iNO, FeNO measurement devices, Nanocarrier delivery systems (future)
Key therapeutic application	Adjunctive COVID-19 therapy (pneumonia, PASC), Hospital disinfection (surfaces, N95), Potential ARDS (O3-MAH)	Bronchopleural fistula repair, Endobronchial tumor resection/hemostasis, Complex airway tumors (APC), Potential microbial load control (CAPPJ)	PPHN, Asthma/COPD monitoring & management (FeNO-guided), Potential ARDS rescue therapy, Potential drug-resistant infections (high-dose iNO)
Diagnostic/monitoring Role	Potential via redox state monitoring (nanoprobes)	Limited direct role; used in biosensing tech for NO detection (nanocomposites)	Yes: FeNO for Th2 airway inflammation (Asthma, COPD)
Antimicrobial effect	Yes (Broad-spectrum, direct disruption)	Yes (Broad-spectrum via CAPPJ active species)	Yes (At high doses, >160 ppm)
Main challenge	Toxicity/Narrow therapeutic window, Standardization (dose, route), Lack of long-term data/high-quality evidence, Environmental vs. Medical distinction	Limited mechanistic data (human/*in vivo*), Need for better diagnostics, Evidence often small-scale, Long-term safety data	Standardization (dose, regimen), Lack of long-term data/objective markers, Integrating genotype, Accessibility/Cost, Delivery/Monitoring precision
Future direction	Precision medicine (biomarkers), Combination therapies (e.g., with LXRs), Integrated risk assessment, Standardization, AI	Multi-omics analysis, AI diagnostics, Improved plasma tech (precision/control), Real-world studies	Precision medicine (biomarkers, AI, genotype), Nanodelivery systems, Portable detectors, Standardization, Multi-center databases
Functional crossover	From disinfectant → Immuno/Redox modulator (O3-MAH)	From inert carrier (cryoablation) → Active therapeutic tool (APC/CAPPJ tissue effects)	From vasodilator (PPHN) → Inflammation monitor (FeNO) & potential antimicrobial

AI, Artificial Intelligence; APC, Argon Plasma Coagulation; ARDS, Acute Respiratory Distress Syndrome; CAPPJ, Cold Atmospheric Plasma Jet; COPD, Chronic Obstructive Pulmonary Disease; ECMO, Extracorporeal Membrane Oxygenation; FeNO, Fractional Exhaled Nitric Oxide; iNO, Inhaled Nitric Oxide; LXRs, Liver X Receptors; Nrf2, Nuclear factor erythroid 2-related factor 2; O3-MAH, Ozone Major Autohemotherapy; PASC, Postacute sequelae of severe acute respiratory syndrome coronavirus 2 (SARS-CoV-2) infection; PPHN, Persistent Pulmonary Hypertension of the Newborn; Th2, T helper 2 cells.

The synergistic effects of these three major gas therapies are already beginning to emerge. Ozone, by activating the Nrf2 pathway and reshaping the microbiome, bridges the gap between managing acute and chronic inflammation. Argon, through the integration of cold plasma technology and nanomaterials, has made a significant leap, advancing from pathogen clearance to tissue repair. NO, as an endogenous signaling molecule, is driving the diagnosis and treatment of asthma and pulmonary hypertension into the “personalized era” through dynamic monitoring and precision delivery technologies. Importantly, the crossover integration of gas therapies has transcended traditional disciplinary boundaries. AI-driven dose optimization models, nanocarrier-based targeted delivery systems, and collaborative management frameworks in both environmental medicine and clinical treatment collectively outline the emerging prototype of “Precision Medicine 2.0.”

Looking to the future, medical gas therapies hold the potential to reshape the broader medical landscape. In disease prevention, early detection platforms leveraging FeNO and oxidative stress biomarkers could enable interventions during the subclinical phase, shifting the focus toward proactive respiratory health management. In critical care, closed-loop systems integrating ECMO with gas-targeted therapies may offer new strategies to overcome the challenges of multi-organ failure. On a public health scale, coordinated approaches that link medical gas technologies with air pollution control efforts may usher in a new era of integrated “environment-to-individual” health management. Realizing this vision will require the global scientific community to embrace interdisciplinary collaboration more fully, fostering an innovative ecosystem that unites basic research, clinical translation, environmental science, and policy development in pursuit of a common goal: improving respiratory health across populations.
